# Congenital Unilateral Corneal Anaesthesia with Microphthalmos: A Case Report

**DOI:** 10.1155/2012/703183

**Published:** 2012-03-27

**Authors:** George Voyatzis, Achyut Mukherjee, Madhavan S. Rajan, Louise E. Allen

**Affiliations:** Ophthalmology Department, Addenbrooke's Hospital, Cambridge University Hospitals NHS Foundation Trust, Cambridge Biomedical Campus, Hills Road, Cambridge CB2 0QQ, UK

## Abstract

Congenital corneal anaesthesia (CCA) is an uncommon condition difficult to diagnose. We report the case of a 20-month-old boy who presented with unilateral congenital corneal anaesthesia. The child was referred with a persistent corneal epithelial defect, unresponsive to symptomatic local treatment for over 10 months. Intensive topical treatment and strict corneal protection led to quick corneal healing. Congenital corneal anaesthesia occurs either alone or in association with neurological diseases or systemic congenital abnormalities. It is important to search for corneal anaesthesia in children with chronic ulcerations of the cornea and self-inflicted injuries. Early diagnosis and treatment are important due to the risk of poor visual prognosis. Management of CCA should aim for the prevention of epithelial defects and is a life-long process.

## 1. Introduction

Congenital corneal anaesthesia (CCA) is a rare clinical entity. The condition usually presents between the age of 6 and 12 months and can pose a diagnostic problem [1]. Infants with this condition need to be promptly identified and treated in order to prevent permanent visual loss due to corneal complications or dense amblyopia [2].We present a rare case of unilateral microphthalmos with CCA where the diagnosis was delayed for more than 10 months. As a result of the visual deprivation, a dense amblyopia was established, which despite the quick resolution of the corneal pathology has proven difficult to treat.

## 2. Case Report

A district ophthalmology department referred a 20 months old boy to our tertiary ophthalmology centre. The child was being treated for 10 months for recurrent central corneal epithelial defect and a persistent inflammation of his right eye. The patient was unresponsive to symptomatic topical treatment with lubricating drops, ganciclovir ointment, and topical steroids.

On arrival the patient had a visual acuity of logMAR 0.6 with Kay Pictures at 50 cm in the left unaffected eye. He objected strongly to left eye occlusion and could not follow a target or make eye contact with his right eye.

There was no family history of note. The child had a forceps delivery with significant bruising over the right eye but was otherwise healthy, and there was no history of chicken pox or any skin blisters or cold sores.

**Figure 1:**
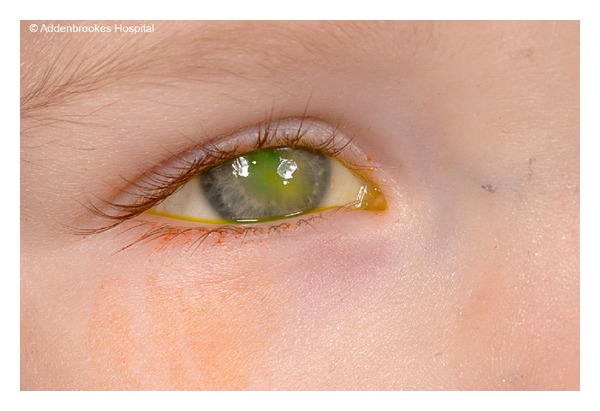
Slit lamp photograph of right eye cornea showing the central persistent epithelial defect measuring 2 × 4 mm with stromal loss.

Questioning his mother revealed that he had a habit of rubbing his right eye with his fingers. On examination there was mild hypoaesthesia of the skin in the ophthalmic division of the trigeminal nerve, there was also corneal and conjunctival anaesthesia of the right eye. The left cornea was normal with no microcysts or any other signs of corneal pathology. Posterior segment examination was normal. Examination under anaesthesia (EUA) showed a 2 × 4 mm central epithelial defect with mild surrounding oedema and moderate anterior chamber activity with a 1 mm hypopyon (Figure 1). 

The right eye was microphthalmic with a horizontal corneal diameter of 10 mm compared to 11.5 mm of the left eye. Refraction revealed +3.0 dioptres of hyperopia in the right eye compared to Plano refraction in the left.

A corneal scrape was performed and plated on blood agar medium for microbiology culture, a conjunctival viral swab was sent for virology tests and a corneal viral scrape was sent for PCR analysis with a special interest in herpes simplex or varicella-zoster viruses. All tests were negative.

Intensive treatment with drops preservative free (PF) levofloxacin 0.5% (Oftaquix, Santen) every 2 hours, ointment aciclovir four times a day, and drops preservative free atropine 1% nocte in the right eye were started. An eye shield to cover the eye and prevent oculodigital injury was provided, and the parents were educated to prevent the patient from rubbing his eye.

Full spectacle correction for his hyperopia was given and occlusion therapy for his right amblyopia was begun.

Over the next 3 weeks, the corneal epithelial defect slowly healed. At four weeks following the EUA, the epithelium was completely healed with a residual faint subepithelial scar and some superficial corneal neovascularisation. All topical medications were stopped and the patient was started on drops (PF) carmellose 0.5% (Celluvisc, Allergan) on a four times a day basis.

The patient remains under followup. He is currently using drops (PF) carmellose 0.5% when needed and wears special spectacles constructed with side shields to prevent self-inflicting corneal trauma.

Six months following his visit his eye remains quiet and fully epithelialized with only a very faint subepithelial scar.

Despite successive attempts for patching the patient could not tolerate it, at best there was limited compliance and there is no improvement in his right visual acuity with the eye remaining densely amblyopic.

## 3. Discussion

Intact corneal sensation is very important for the integrity of the corneal epithelium. It prevents injury through the blink reflex, it lubricates the ocular surface with reflex tearing, and it promotes the healing of epithelial defects. Any sensory deficit of the cornea increases the risk of epithelial breakdown, persistent epithelial defects, and corneal infection [3].

Congenital corneal anaesthesia is a rare clinical entity, which can cause diagnostic problems in the paediatric population. Congenital corneal anaesthesia can be misdiagnosed as herpes virus keratitis, dry eyes, or recurrent epithelial erosions.

It can remain asymptomatic and only be diagnosed after careful examination or it may present as persistent corneal ulceration refractory to treatment. It can be bilateral or, rarely, unilateral and usually presents between the ages of 6 to 12 months.

The clinician should be suspicious off any child presenting with recurrent unilateral or bilateral episodes of conjunctival hyperaemia, photophobia, or corneal ulcers that are not accompanied by pain or distress [4, 5]. Neurotropic infections, such as herpes simplex keratitis, causing corneal hypoaesthesia need to be excluded. Brain neuroimaging to exclude the possibility of a rare cerebellopontine tumour should also be considered.

Treating congenital corneal anaesthesia is life-long and should focus on the detection and treatment of associated systemic diseases, the healing of the cornea, the prevention of future ocular trauma, and the treatment of amblyopia.

In our case the patient was treated for more than 10 months symptomatically for presumed recurrent corneal erosions without an established diagnosis. During this period he developed a dense amblyopia that persists despite the rapid healing of his cornea during this period he developed a dense amblyopia and presently he is undergoing a patching regime.

Accurate identification and prompt treatment of this rare condition is of paramount importance to prevent chronic corneal changes and long-term visual loss.
